# Editorial: Neurocognitive features of human-robot and human-machine interaction

**DOI:** 10.3389/fpsyg.2024.1394970

**Published:** 2024-03-14

**Authors:** Francesco Bossi, Francesca Ciardo, Ghilès Mostafaoui

**Affiliations:** ^1^Department of Information Engineering, University of Pisa, Pisa, Italy; ^2^Department of Psychology, University of Milano Bicocca, Milan, Italy; ^3^ETIS Laboratory, CY Cergy Paris University, ENSEA, CNRS, Cergy-Pontoise, France

**Keywords:** EEG, Human-Robot Interaction (HRI), Human-Computer Interaction (HCI), conversational agent (CA), brain computer interface (BCI), autism spectrum conditions (ASC), steady-state visual evoked potential (SSVEP)

The growing integration of machines and robots in different contexts of our daily lives is becoming increasingly apparent. Despite notable advancements, the capability of machines and robots to engage humans naturally and intuitively remains inadequate. Therefore, this Research Topic investigates the “human factor” while interacting with artificial agents from both behavioral and neuroscientific perspectives. We gathered studies analyzing humans interacting with different types of machines encompassing physical robots, computer interfaces, conversational agents (CA) and brain-computer interfaces (BCI) settings.

Regarding Human-Robot Interactions (HRI), one crucial question is to what extent robots are spontaneously perceived and treated as intentional agents. In their work, Morillo-Mendez et al. addressed it by adopting a neurocognitive-inspired approach (Wiese et al., 2017). The authors analyzed to what extent humans attribute intentionality toward a robot by testing the gaze-following behavior. Results showed that participants followed the gaze of the robot when they believed the NAO robot could potentially see the target. Interestingly, the gaze-following behavior was attenuated when, according to the participants' point of view, the NAO robot could not see the target. The study by Morillo-Mendez et al. suggests that gaze following with an anthropomorphic robot occurs if humans can attribute a mental state toward it. This study is embedded in a new series of studies aiming to use robots to better understand human behaviors (Wykowska, 2020). The work by Mondellini et al. is an example of such an approach. The authors adopted an ecological approach to evaluate, in HRI, differences in human performance by comparing adults with autistic syndrome condition (ASC) and neurotypical ones (NT). They analyzed both qualitative and quantitative behavioral patterns during an assembly task in an industry-like lab-based robotic collaborative cell. Quantitative results showed that most of the participants with ASC displayed longer waiting times for the robot compared to NT, suggesting a lower sense of urgency in attending to the robot. Also, NT participants spent more time looking at the robot compared to the participants with ASC. Taken together, these results highlight how individual differences are crucial factors in the design of cobots. Exploring these aspects provides valuable insights into how individuals with different needs might cope with HRI.

When considering virtual artificial agents, the field of Human-Computer Interaction (HCI) has seen a growing interest related to the interaction with conversational agents (CA) and how they can influence the user experience (Moussawi et al., 2021). Poivet et al. developed a computer textual game in which participants endorse the role of a detective and have to discuss with four different CAs which could have different roles (witness or suspect) and communication styles (aggressive vs. cooperative). The role and communication style influenced participants' evaluation of the CA in terms of intelligence and believability. Specifically, CAs' communication styles played a crucial role in shaping participants' perception of aggressiveness and warmth and influenced also participants' behaviors. The authors conclude that aligning the roles of CAs with their communication styles has the potential to significantly improve users' experience and that taking into account parameters such as CA's roles, communication styles, and user expectations can enhance the immersive and interactive nature of the narrative.

Today, AI is also extensively adopted in automated journalism for writing and broadcasting (Heiselberg et al., 2022). Yet, there has been limited systematic research on the variances in brain activation between human and artificial voices during newscasts. The study by Gong explored the psychophysiological effects of media in Chinese settings. Gong compared the electroencephalography (EEG) signals of adults while exposed to various newscast agents (Human or AI). Results showed that greater β band activity was observed in the left posterior temporal lobe when participants listened to a human newscast compared to an AI voice newscast, indicating that participants' brains exhibited enhanced processing when listening to a human reporter rather than an AI-generated voice.

Another important application of HCI is the brain-computer interface (BCI), i.e., a direct communication pathway between the brain and an external device or computer system (Vaid et al., 2015). BCIs allow for the exchange of information between the brain and external devices, typically by analyzing, classifying and decoding EEG signals. The steady-state visual evoked potential (SSVEP) is a type of brain signal that occurs in response to repetitive visual stimuli presented at a constant frequency and it is crucial in the implementation of BCI systems. A significant challenge with SSVEP is that most approaches do not effectively handle non-stationary and time-varying EEG signals. In their study, Yan et al. addressed this issue by introducing an unsupervised adaptive classification algorithm founded on the self-similarity of signals at the same frequency. The proposed algorithm ensures continuous updating of template signals as new EEG data is inputted, which is crucial in real-time BCI. The authors showcased the efficacy of leveraging the self-similarity of signals at the same frequency within the adaptive classification algorithm.

In conclusion, critical advancements were recently made in understanding different aspects of the human factor in interaction with several types of artificial agents, shedding light on the benefit of adopting an interdisciplinary approach in the broad field of social interactions with artificial agents. Nevertheless, these advancements opened new questions that will need to be answered in future research, given the paramount role that AI and robots are taking in our daily lives.

## Author contributions

FB: Writing – review & editing, Writing – original draft. FC: Writing – review & editing, Writing – original draft. GM: Writing – review & editing, Writing – original draft.
